# Wall‐following – Phylogenetic context of an enhanced behaviour in stygomorphic *Sinocyclocheilus* (Cypriniformes: Cyprinidae) cavefishes

**DOI:** 10.1002/ece3.11575

**Published:** 2024-06-25

**Authors:** Bing Chen, Wen‐Zhang Dai, Xiang‐Lin Li, Ting‐Ru Mao, Ye‐Wei Liu, Marcio R. Pie, Jian Yang, Madhava Meegaskumbura

**Affiliations:** ^1^ Guangxi Key Laboratory for Forest Ecology and Conservation, College of Forestry Guangxi University Nanning China; ^2^ Ministry of Education Key Laboratory for Biodiversity Science and Ecological Engineering, Institute of Biodiversity Science, Center of Evolutionary Biology, School of Life Sciences Fudan University Shanghai China; ^3^ School of Life Science and Institute of Wetland Ecology Nanjing University Nanjing China; ^4^ State Key Laboratory of Efficient Production of Forest Resources School of Ecology and Nature Conservation, Beijing Forestry University Beijing China; ^5^ Biology Department Edge Hill University Ormskirk Lancashire UK; ^6^ Key Laboratory of Environment Change and Resource Use, Beibu Gulf Nanning Normal University Nanning Guangxi China

**Keywords:** animal tracking, evolutionary convergence, exploratory behaviour, phylogeny, stygobitic, wall‐following

## Abstract

With 75 known species, the freshwater fish genus *Sinocyclocheilus* is the largest cavefish radiation in the world and shows multiple adaptations for cave‐dwelling (stygomorphic adaptations), which include a range of traits such as eye degeneration (normal‐eyed, micro‐eyed and eyeless), depigmentation of skin, and in some species, the presence of “horns”. Their behavioural adaptations to subterranean environments, however, are poorly understood. Wall‐following (WF) behaviour, where an organism remains in close contact with the boundary demarcating its habitat when in the dark, is a peculiar behaviour observed in a wide range of animals and is enhanced in cave dwellers. Hence, we hypothesise that wall‐following is also present in *Sinocyclocheilus*, possibly enhanced in eyeless species compared to eye bearing (normal‐/micro‐eyed species). Using 13 species representative of *Sinocyclocheilus* radiation and eye morphs, we designed a series of assays, based on pre‐existing methods for *Astyanax mexicanus* behavioural experiments, to examine wall‐following behaviour under three conditions. Our results indicate that eyeless species exhibit significantly enhanced intensities of WF compared to normal‐eyed species, with micro‐eyed forms demonstrating intermediate intensities in the WF distance. Using a mtDNA based dated phylogeny (chronogram with four clades A–D), we traced the degree of WF of these forms to outline common patterns. We show that the intensity of WF behaviour is higher in the subterranean clades compared to clades dominated by normal‐eyed free‐living species. We also found that eyeless species are highly sensitive to vibrations, whereas normal‐eyed species are the least sensitive. Since WF behaviour is presented to some degree in all *Sinocyclocheilus* species, and given that these fishes evolved in the late Miocene, we identify this behaviour as being ancestral with WF enhancement related to cave occupation. Results from this diversification‐scale study of cavefish behaviour suggest that enhanced wall‐following behaviour may be a convergent trait across all stygomorphic lineages.

## Graphical abstract

*Sinocyclocheilus*, a genus of 75 species of freshwater cavefish, is an emerging model system in evolutionary studies. We studied a behaviour, called “wall‐following,” where fish stay close to the cave walls in absence of light. We show that eyeless species exhibited heightened wall‐following behaviour compared to their sighted counterparts, with small‐eyed species falling in between. We also mapped this behaviour on a phylogenetic tree, discovering a pattern where cave‐dwelling clades showed stronger wall‐following than free‐living ones. Wall‐following is prevalent in all *Sinocyclocheilus* species and, given the evolutionary history of the genus, is considered an ancestral behaviour that intensified with cave adaptation.

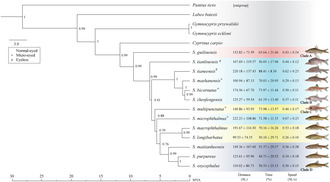

## 1 INTRODUCTION

Vertebrate lineages have evolved sensory systems and associated behaviours in order to adapt to new environments such as subterranean habitats. To occupy caves, species became adapted to the low availability of resources such as light, oxygen concentration and nutrients, leading to stygomorphic adaptations, including elongated appendages, lowered metabolism, specialised sensory systems, loss of eyes and pigmentation (Chen et al., 2020; Jeffery, 2019; Li et al., 2020; Ma, Gore, et al., 2020; Yoshizawa et al., 2012). A prominent stygomorphic convergent feature of cavefishes is the degeneration of eyes, compensated for by enhancements to the mechanosensory organs such as the neuromast lateral line system (Borowsky, 2013; Chen, Mao, et al., 2022; Ma, Herzog, et al., 2020). A prominent swimming behaviour of cavefish is wall‐following (WF, a form of thigmotaxis), where the fish senses the walls or boundaries of its cave environment in the absence of visual cues (Patton et al., 2010; Sharma et al., 2009). Although thigmotaxis has been reported in non‐cavernicolous organisms introduced into a dark environment, this behaviour is putatively enhanced in cave dwellers (Niemiller & Soares, 2015; Norton, 2012; Sharma et al., 2009).

Wall‐following behaviour has previously been observed in freshwater fish such as *Astyanax mexicanus*, *Gasterosteus aculeatus* and *Danio rerio* (Ginnaw et al., 2020; Johnson & Hamilton, 2017; Patton et al., 2010). A lion's share of this work has been on *A. mexicanus*, where some populations are cave‐dwelling and exhibit distinct adaptations for cave life. Some eyeless populations are capable of moving through complex environments without colliding with objects and their larvae prefer using a frontal approach using their head (Lloyd et al., 2018). Hence, cavefish resort to continuous swimming in order to constantly receive information from the environment (Holbrook & de Perera, 2013; Windsor et al., 2008). Once cavefish detect a cave wall, they continue following the wall (Patton et al., 2010), which suggests that wall‐following in cavefish is both spontaneous and continuous. This behaviour is enhanced in cavefish compared to other animals. Past studies have proposed wall‐following behaviour as a strategy for foraging and spatial exploration (Sharma et al., 2009). In their perpetually dark environments, cavefish have evolved better short‐range senses (hydrodynamic imaging ability) (Hassan, 1985; Windsor, 2014), such as tactile sensing using the anterior part of their body (Sharma et al., 2009) and using mouth suction frequently to generate a suction flow to navigate non‐visually (Holzman et al., 2014). This implies that wall‐following might entail complex functions such as spatial orientation, seeking protection or refuge and obstacle avoidance.

Distinguishing between stationary and moving objects is of vital importance for cavefish due to the limited sensory perception in short distances within restricted cave environments. A study in *A. mexicanus* showed differences in WF behaviours under different boundary stimulations, observing that eyeless morphs swim nearly parallel to the wall, compared to sighted morphs. Under varying light conditions, eyeless morphs expressed a closer swimming distance to the wall while reaching a higher swimming speed compared to sighted morphs (Sharma et al., 2009). However, the evolution of wall‐following behaviour (WF) in response to vision loss (morphological change) remains poorly understood.

With 75 species, the genus *Sinocyclocheilus* (Cyprinidae, Barbinae) represents the largest cavefish radiation in the world (Jiang et al., 2019; Mao et al., 2021). These species show substantial morphological variability and inhabit suitable habitats of the massive 62,000 km^2^ south‐western karstic landscape of China (Jiang et al., 2019; Romero et al., 2009; Xiao et al., 2005; Zhao & Zhang, 2009). They are phylogenetically well known, with four major clades (A–D), with clades B & C harbouring mostly the stygomorphic forms and clades A & D containing predominantly the surface‐dwelling forms (Mao et al., 2021). They represent an emerging model system for evolutionary novelty and show multiple adaptations for subterranean life. For instance, they demonstrate varying degrees of eye degeneration, from normal‐eyed to micro‐eyed and eyeless (Meng et al., 2013; Zhao et al., 2021), loss of pigmentation (Li et al., 2020; Luo et al., 2023), absence of circadian rhythms, slow metabolism (Yang et al., 2016; Zheng, 2017) and a horn‐like structure on the head on some species, of which the function is not clear (He et al., 2013). Studies on their natural history suggest that Eyeless cavefish are less active than Normal‐eyed species. For instance, *S*. *grahami* (Normal‐eyed, surface‐dwelling) swim faster and farther, swimming at a speed 2–3 times greater than *S. anshuiensis* (Zheng, 2017). The neuromast system in *Sinocyclocheilus* has been shown to be asymmetric, is correlated with the degree of eye degeneration and is pronounced in the eyeless forms (Chen, Mao, et al., 2022). Furthermore, for several species, the WF behaviour is also thought to be associated with neuromast asymmetry, with eyeless forms having the strongest WF behaviours (Chen, Li, & Madhava, 2022). Deeper exploration is needed to understand the phylogenetic context and elucidate the range of stimuli influencing WF behaviour. Furthermore, a study of *Sinocyclocheilus* showed eyeless morphs being attracted to different stimuli (Chen, Li, & Madhava, 2022). Given the large number of species, the genus *Sinocyclocheilus* offers an ideal system for an in‐depth analysis of behaviour across a cavefish radiation.

Despite being an emerging multi‐species model system, a radiation‐scale understanding of *Sinocyclocheilus* cavefishes' swimming behaviour is still lacking. Here, we investigate the swimming behaviour of *Sinocyclocheilus* species in a phylogenetic context. The species considered represent the three main habitat types and the three main eye‐related morphologies: Normal‐eyed (surface water bodies, surface‐dwelling habit), Micro‐eyed (cave‐associated habitats, stygophilic habit) and Eyeless (cave habitat, stygobitic habit) in the context of Mao et al. (2021). Given that WF behaviour is arguably enhanced in *A. mexicanus* cavefish populations and it is potentially affected by the sensory organs (e.g. neuromasts in the lateral line system), we hypothesise that in *Sinocyclocheilus* species, WF is a shared, derived trait correlated with visual acuity – a characteristic for which we use eye morphs as a proxy. Hence, we predict that eyeless species will show the greatest intensity of WF behaviour, followed by micro‐eyed and normal‐eyed species, respectively. Furthermore, we predict that various (stable/vibrative) stimuli will elicit a distinct response correlated to the extent of eye degeneration condition.

## 2 MATERIALS AND METHODS

### 2.1 Fish collection and maintenance

**FIGURE A1:**
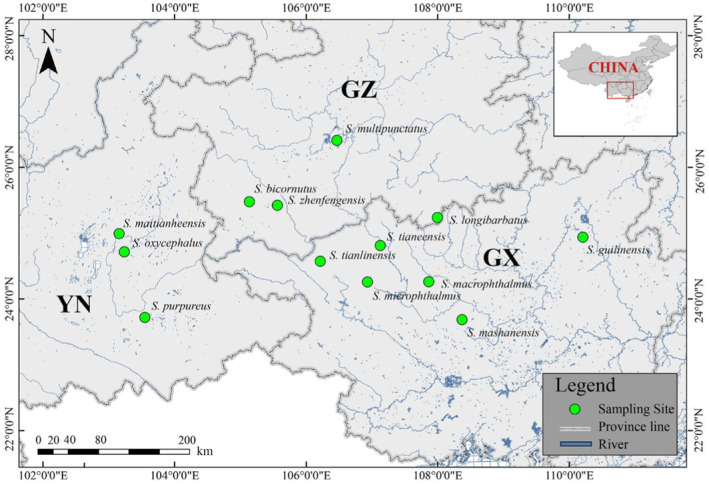
Map of the sampling sites of the 13 species of *Sinocyclocheilus* (*N* = 39).

All experimental fishes (13 *Sinocyclocheilus* species, with 3 individuals from each species, *N* = 39) were adults (Standard Length, SL: mean ± SD = 8.40 ± 1.28 cm) and collected from Yunnan and Guizhou Province and Guangxi Zhuang Autonomous Region of China between December, 2017 and September, 2020 (Table A1, Figure A1). Fish smaller than 8 cm were maintained in a Centralised Zebrafish aquarium system and housed in 1 L BPA‐free plastic tanks with each tank receiving separate water delivery and drainage. Fish larger than 8 cm were maintained in groups in four large aquariums (90 × 50 × 50 cm, 300 L; 150 × 80 × 80 cm, 1000 L capacity), equipped with dedicated filtration and purification equipment. Fish were regularly maintained on Shenyangkangcai™ fish food every day, consisting of shrimp, squid, spring fish and seaweed. We classified the species according to gross eye morphology as follows: Normal‐eyed – *S. guilinensis*, *S. zhenfengensis*, *S. longibarbatus*, *S. macrophthalmus*, *S. oxycephalus*, *S. purpureus*, *S. maitianheensis*; Micro‐eyed – *S. mashanensis*, *S. microphthalmus*, *S. bicornutus*, *S. multipunctatus*; and Eyeless – *S. tianlinensis*, *S. tianeensis*. Based on the variations in craniocaudal axis and dorso‐ventral axis, we designated the body shape as: fusiform (longer craniocaudal axis) – *S. macrophthalmus*, *S. oxycephalus* (Li, 1985; Wang & Chen, 1989); others were all categorised as compressiform (relative longer dorso‐ventral axis) (Zhao & Zhang, 2009) (Table A1).

### 2.2 Experimental equipment and video recording

**FIGURE 1:**
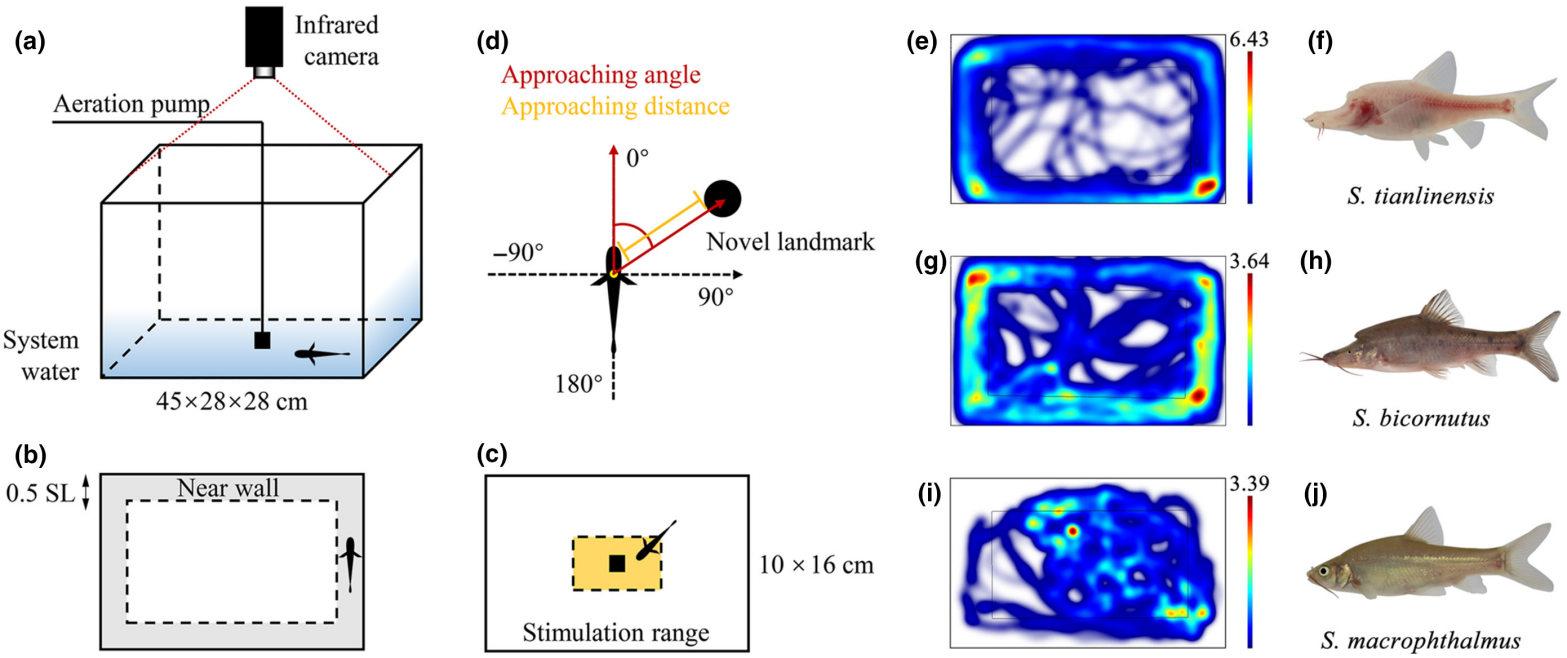
Diagram of the experimental apparatus and schematic diagram of measurements the representative trajectories of three species in the 10 min assay. (a) Diagram of the experimental tank and equipment. (b) Vertical view of wall‐following assay. The wall‐following range is shown as the grey area, while its width is 0.5 SL. (c) Novel landmark and VA assay's stimulation range are shown in yellow. (d) Approaching angle and distance used for stimulation‐approaching analysis. (e, g, i) Representative swimming trajectories for the three forms: *Sinocyclocheilus tianlinensis* (eyeless, stygobitic, fusiform), *S. bicornutus* (micro‐eyed, stygophilic, fusiform) and *S. macrophthalmus* (normal‐eyed, surface, compressiform) in 10 min with no stimulation assay's distance to the “near wall”. The colours represent the duration the fish spent in each pixel with low wavelengths (red) indicating greater times spent and high wavelengths (blue) indicating lower times spent at each pixel. Eyeless and Micro‐eyed species maintain wall‐following for a longer duration than the Normal‐eyed species. Trajectory charts were created using the EthoVision XT software. (f, h, j) The three species for which the behaviour is depicted here.

For all assays, an individual was tested in one 45 × 28 × 28 cm rectangular assay arena. We used the aquarium system water (pH: 7.0–8.0, conductivity 150–300 S/m, temperature 19 ± 1°C, dissolved oxygen 8.5 mg/L), with water quality simulating natural conditions as much as possible. The depth of water was shallow (10–15 cm) depending on the size of the fish, aiming to reduce their vertical excursions and to minimise depth of field errors with the movement tracking system (as explained below). To minimise stress factors associated with differences in water properties, we changed the system water in the tank after each assay. The fish were allowed a minimum of 10 min to acclimate and recover from the transfer process. Subsequently, the infrared illumination and digital video camera were activated. An infrared camera (Cannon XF 405) was set up about 1 m above the tank. An auxiliary infrared light source (850 nm; HongGuang, HG‐IR1206, GangDong) uniformly irradiates the assay arena. We used a 4 Mbps (YCC 4:2:0, 25p, 1280 × 720) system setting to capture video under the infrared light (Figure 1a). The experimental design also followed the methods outlined by Chen, Mao, et al. (2022). Due to our inability to visually track the cavefish in complete darkness, and in order to enhance the precision of software tracking, we adjusted our experimental settings. Noting that species with eyes ceased movement in total 0 Lux environments, all assays were conducted in a quiet, nearly dark room (1.7–5 Lux). We repeated each trial 3–10 times for each *Sinocyclocheilus* species under identical conditions. After the conclusions of the experimental trials, we checked the results and removed the videos in which cavefishes were completely stationary or could not be analysed by the limitations of the software. The results of each individual utilised in the model were designated as a random effect in the analysis (see Section 2). Except for when gravid, *Sinocyclocheilus* cavefishes do not show sexual dimorphism (Zhao & Zhang, 2009); together with their given rarity, we did not consider gender‐related behavioural differences. All individuals survived the experimentation.

### 2.3 Wall‐following assays

Since wall‐following behavioural assays are established for *A. mexicanus* (Patton et al., 2010; Sharma et al., 2009; Windsor et al., 2008), we followed these in our study. We defined wall‐following behaviour as swimming along the wall within a distance of 0.5 SL, and this area was called the near‐wall belt (range of wall‐following, Figure 1b). When fish maintained travelling a minimum distance for 2 SLs within the near‐wall belt, we recorded it as wall‐following behaviour. We used the EthoVision XT v.15 (Noldus IT, Wageningen, Netherlands) to track the swimming trajectories (Figure 1e,g,i). Standard Length (SL) and pectoral fin length (PFL) were measured using FIJI (https://imagej.nih.gov) (Schneider et al., 2012). Swimming speeds less than 0.2 cm/s were set as immobility (resting) when analysing with EthoVision XT. We generated data for the following indicators in the near‐wall belt: WF‐Distance (the distance fish swimming past within the wall‐following range), WF‐Frequency (the frequency at which fish swam into wall‐following range), WF‐Time (the time that fish spent wall‐following), WF‐Resting Time (the resting time during one assay), WF‐Speed (average fish swimming speed when wall‐following) and WF‐Max Speed (the maximum speed fish reached during one assay; Table A1; abbreviations summarised in Table A2).

**FIGURE 2:**
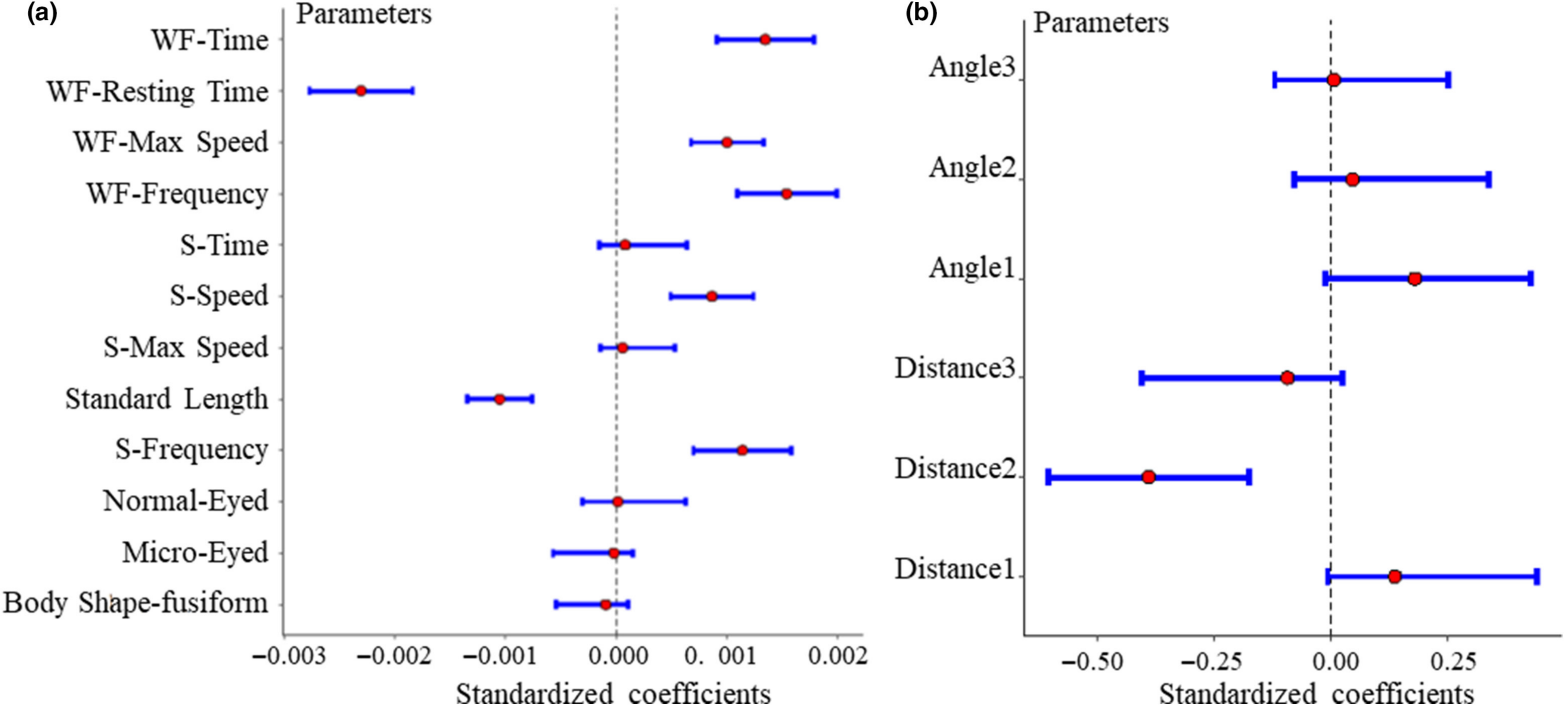
The effect of wall‐following measurement parameters, with 95% confidence intervals, on the Wall‐following distance of assaying *Sinocyclocheilus* species (a) and the results of approaching angle/distance related with stimulation (b).

### 2.4 Quantification of reaction to stimuli

To understand whether wall‐following is a fixed behaviour or whether it could be affected by various stimuli, we performed assays under three different stimuli: one without stimulation, one with a novel landmark and one with a vibration attraction (VA) setting. First, the unimpeded forward motion along the wall for 10 min was observed to figure out whether WF behaviour occurs (Video S1). Second, we tested for responses to a novel landmark for 5 min by placing a dark opaque cylinder (diameter = 5 cm) in the centre of a rectangular arena, following the methods outlined in Burt de Perera and Braithwaite (2005) and Lloyd et al. (2018) (Video S2). Third, we assayed vibration attraction stimulation for 3 min, following the methods outlined in Jiang et al. (2019) and Fernandes et al. (2018) (Video S3). Vibrations were produced with an aeration pump (Jialu, LT‐201S™, ZheJiang) working at 40–50 Hz placed in the centre of the arena. The 10 × 16 cm rectangular area around the stimulation was considered as the stimulation range (Figure 1c). We measured the following indicators of fish swimming within this range: S‐Frequency (the frequency at which fish swam in the stimulation range), S‐Time (the time fish spent in the stimulation range), S‐Speed (the average fish swimming speed) and S‐Max Speed (the maximum speed they reached during an assay; Table A2).

To distinguish how various stimuli affect the behaviour of *Sinocyclocheilus* cavefish, we also measured two additional indicators: the angle of approach (Approaching angle) and the distance of approach (Approaching distance) to stimulation, which were quantified using FIJI. Approaching distance was defined as the shortest distance between the edge of the fish's body and the stimulation (Figure 1d). The approaching angle was defined as the angle between a line extending down the midline of the fish and a line extending from the centre of stimulation again, following protocols established by Lloyd et al. (2018). In each assay, we recorded only the first three repeated approaches. Numbers from 1 to 3 represented the order of approaching within a given assay. For instance, “Angle 1” and “Distance 1” indicated the first time the fish were attracted by the stimulation.

### 2.5 Analysing the behaviour in an evolutionary context

Previous studies have not provided definitive explanations regarding whether wall‐following behaviour is an adaptation to cave environments or if it is associated with ancestral cave preferences (Patton et al., 2010; Sharma et al., 2009). Hence, we further analysed the evolution of wall‐following behaviour from a phylogenetic perspective. We obtained two mtDNA fragments (Cytb and ND4) of 13 *Sinocyclocheilus* species and 5 outgroup species: *Cyprinus carpio*, *Puntius ticto*, *Labeo batesii*, *Gymnocypris przewalskii* and *Gymnocypris eckloni* from Genbank (Table A1). We edited and aligned sequences using Mega 6 (Tamura et al., 2013). BEAST v1.8.4 (Suchard et al., 2018) was implemented to build a Bayesian chronogram using two fossil calibration points (C1, C2) as recommended by Li et al. (2008). Optimal substitution model and parameter settings were determined by jModeltest (Darriba et al., 2012). We then mapped behavioural patterns onto the inferred tree to assess their evolutionary trajectories.

### 2.6 Statistical analysis

All the analyses were conducted in R v4.1.0 (R Core Team, 2021). Given that interspecific trait variation is often confounded by phylogenetic autocorrelation, traditional statistical methods might be subject to biases, such as elevated false‐positive rates, thus requiring specific methods such as phylogenetic generalised least squares (PGLS) (Garamszegi, 2014) regression analysis. We tested for phylogenetic signal in different traits (WF distance, WF speed, SE diameter (eye trait, Table 1, Tables A2 and A3), WF frequency, WF time and WF‐max speed), which was estimated using Pagel's *λ* parameter in package “caper” (Freckleton et al., 2002; Orme et al., 2013). A result such as the value of *λ* nearing 1 denotes a stronger signal (Blomberg et al., 2003; Pagel, 1999). Given that there was no evidence of phylogenetic signal in tested traits (*λ* = 0.000; Table A3), we were justified in using non‐phylogenetic statistical methods in all remaining analyses.

**TABLE 1:** The results of model showing the variables that influenced WF distance and the approaching angle/distance related with stimulation.

	Parameters	Average estimate	Adjusted SE	CI 2.5%	CI 97.5%
WF distance $R_{m}^{2}$ = 0.67 $R_{c}^{2}$ = 0.89	**WF‐Frequency**	**0.0015**	**0.0002**	**0.0011**	**0.0020**
	**WF‐Time**	**0.0014**	**0.0002**	**0.0009**	**0.0018**
	**S‐Frequency**	**0.0011**	**0.0002**	**0.0007**	**0.0016**
	**WF‐Max speed**	**0.0010**	**0.0002**	**0.0007**	**0.0013**
	**S‐Speed**	**0.0009**	**0.0002**	**0.0005**	**0.0012**
	S‐Time	0.0002	0.0002	−0.0002	0.0006
	S‐Max Speed	0.0002	0.0002	−0.0001	0.0005
	Normal‐eyed	0.0002	0.0002	−0.0003	0.0006
	Micro‐eyed	−0.0002	0.0002	−0.0006	0.0002
	Body Shape – fusiform	−0.0002	0.0002	−0.0005	0.0001
	**Standard Length**	**−0.0011**	**0.0002**	**−0.0013**	**−0.0008**
	**WF‐Resting Time**	**−0.0023**	**0.0002**	**−0.0028**	**−0.0018**
Stimulation $R_{m}^{2}$ = 0.15 $R_{c}^{2}$ = 0.15	**Angle1**	**−0.0921**	**0.1100**	**−0.6054**	**−0.1751**
	Angle2	0.0472	0.0949	−0.0770	0.3400
	Angle3	0.1815	0.1127	−0.1148	0.4304
	Distance1	0.1390	0.1148	−0.0061	0.4440
	**Distance2**	**−0.3903**	**0.1064**	**−0.6054**	**−0.1751**
	**Distance3**	**−0.3903**	**0.1098**	**−0.6054**	**−0.1751**

*Note*: Coefficients calculated using the averaged modelled estimates (full averaging technique) method, as well as the associated adjusted SE for the coefficient. The 95% confidence interval (CI) of variables that do not bound 0 are in bold. The number from 1 to 3 represented the order of approaching Angle and Distance to the stimulation. Bold size showed the parameters with significantly differences.

We included the individual fish as a random effect to account for the repeated measures of each fish. To account for individual differences, WF‐Distance and WF‐Speed were expressed in terms of SL and WF‐Time was calculated as the percentage of testing time (%). We formulated generalised linear mixed models (GLMM) to understand the effects of association and coefficients in wall‐following behaviour and stimulation‐approaching behaviour (two separate models) in 13 *Sinocyclocheilus* species by using the R package “lme4” (Bates et al., 2014; Nakagawa et al., 2017). As fixed independent variables, we used fish morphology (Eye‐morphs, Body shape, SL and PFL), wall‐following measurements (WF‐Frequency, WF‐Time, WF‐Resting Time, WFSpeed and WF‐Max Speed) under: 10, 5 and 3 min assays; and the stimulation range behaviour measurements (S‐Frequency, S‐Time, S‐Speed and S‐Max Speed). WF‐Distance was set as the response variable. Testing time was used as a random independent variable. For the model analysing approaching behaviour, we used approaching angle/distance as independent variables and stimulation as response variable. Since the data were diagnosed as over‐dispersion, we selected negative binomial distributions for the final models (Boswell, 1979). We used the package “MuMIn” to assess the AIC (Bartoń, 2019; Burnham & Anderson, 2002). We reported the fully averaged results of the models as a ΔAICc threshold of 2. If the 95% confidence interval of a parameter is higher than zero, we consider it as an important factor in explaining this model (Di Stefano, 2004; Nakagawa & Cuthill, 2007).

## 3 RESULTS

All *Sinocyclocheilus* cavefishes displayed a stereotypic “thigmotaxis” response to the new environment, revealed by an initial preference for wall‐following of the tank. Individual species exhibited variation in their responses to unfamiliar and new environments, such as sudden changes in motionlessness or swimming at a significantly faster pace. These variations possibly highlight the species‐specific adaptations and behavioural strategies that come into play when encountering novel surroundings. However, the nature of wall‐following behaviour differed between species, depending on the eye morphs.

### 3.1 Variables correlated with wall‐following behaviour

WF‐Frequency, WF‐Time, S‐Frequency, WF‐Max Speed and S‐Speed were the important variables affecting WF‐Distance in *Sinocyclocheilus* species (Figure 2a, Table 1). Our results suggested that fish's standard length (SL) and WF‐Resting Time were negatively correlated with WF‐Distance, while WF‐Frequency, WF‐Max Speed, WF‐Time, S‐Frequency and S‐Speed, were positively correlated with WF‐Distance (Figure 2a). Our WF‐Distance and stimulation model has $R_{\text{marginal}}^{2}$ values as 0.67 and 0.15, respectively. We checked the spatial autocorrelation and found none. Only the second approaching distance (Distance2) influenced stimulation negatively (Figure 2b, Table 1). We found that all *Sinocyclocheilus* approached the stimulation at a narrow angle (<90°; Table A4). However, the approaching distance was significantly greater in surface fish than in cavefish (mean ± SD: Eyeless = 0.72 ± 0.91; Micro‐eyed = 1.26 ± 1.07; Normal‐eyed = 1.35 ± 1.08 SL), which suggested an ability to detect unknown objects at longer distances in Normal‐eyed species.

### 3.2 Wall‐following ability is related to eye‐morphs in *Sinocyclocheilus*

**FIGURE 3:**
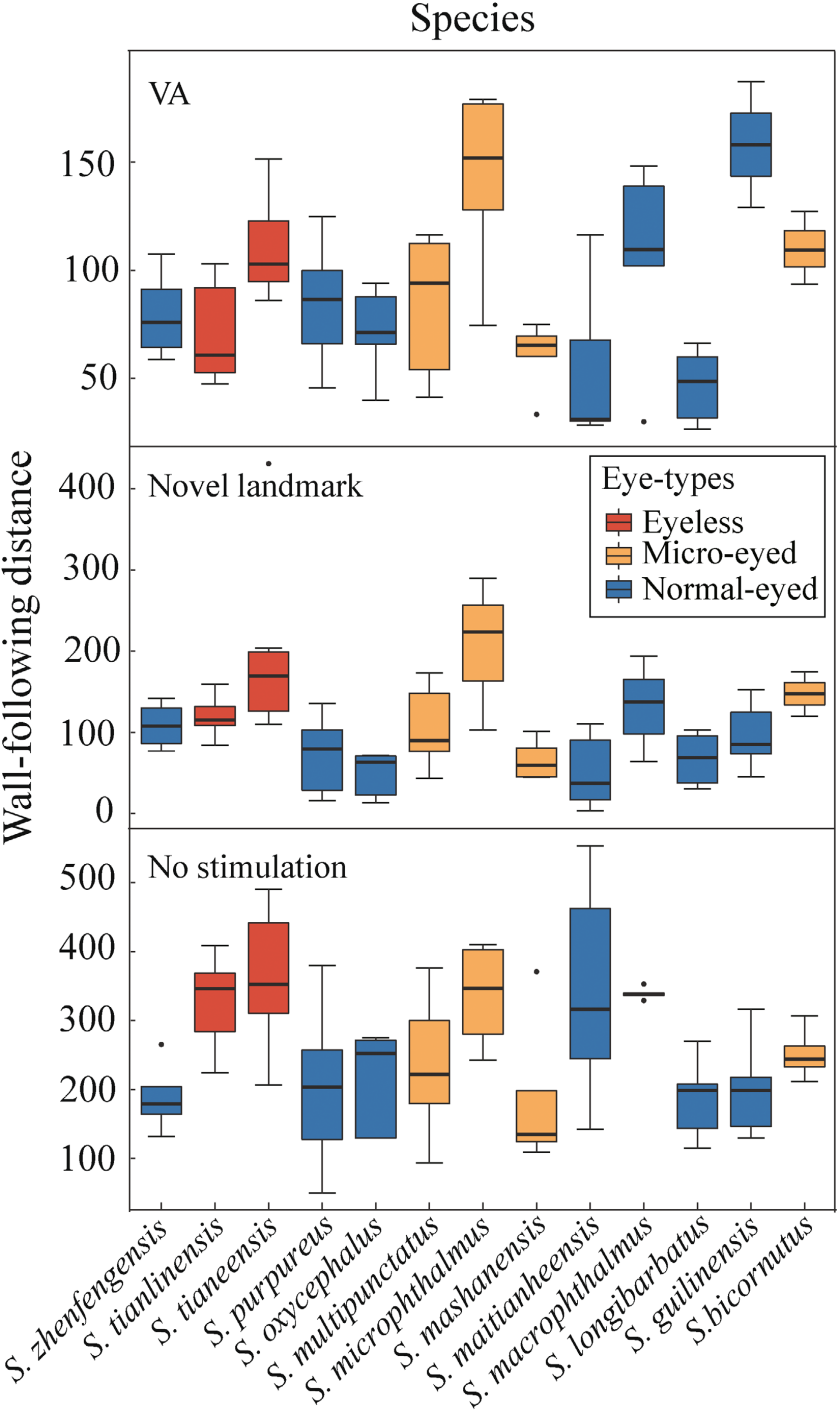
The results of wall‐following distance of 13 *Sinocyclocheilus* species across three tests. Ten minutes with no stimulation, 5 min with novel stimulation and 3 min with vibration attraction behaviour (VA). Different colours on boxes indicate the Eyeless, Micro‐eyed and Normal‐eyed morphs. The wall‐following distance in eyeless groups were significantly higher than in Normal‐eyed groups. Wall‐following distances are expressed in terms of standard length (SL). Details of the statistical analyses are available in Table A5.

**FIGURE 4:**
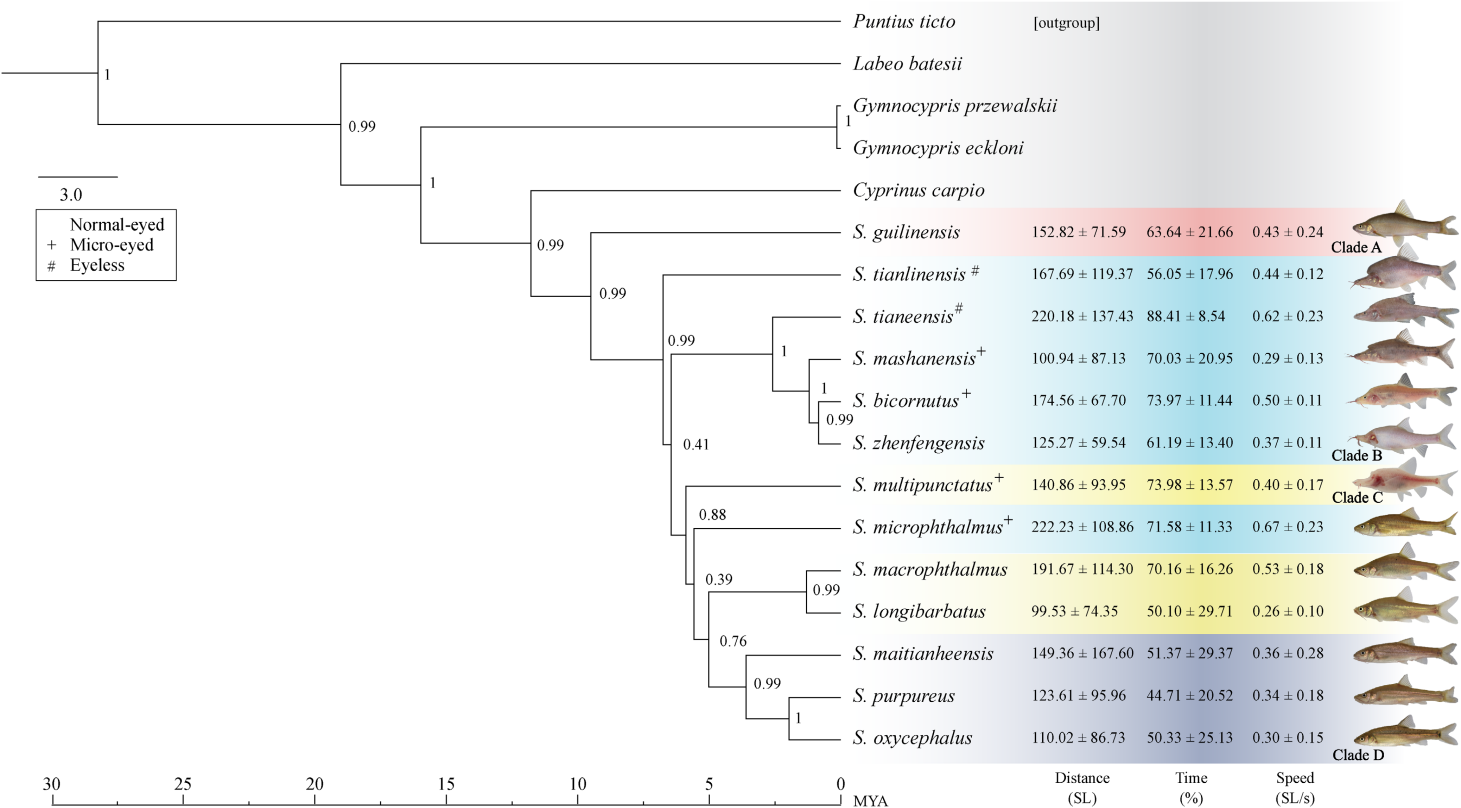
Bayesian inference tree derived from the concatenated data of mt‐DNA and the mean results calculated from 10 mintues' assay. Node values indicate clade posterior probability. Clade A–D are represented by four colours: Clade A – red, Clade B – blue, Clade C – yellow and Clade D – purple. The number represents the distance (SL), time (%) and speed of wall‐following behaviour (SL/s), together with pictures of each species.

We found wall‐following behaviour was ubiquitous across *Sinocyclocheilus* cavefishes but with clear patterns associated with the eye‐morphs. Considering the species studied here, except *S. purpureus*, more than 50% of the time was spent following the wall (Figure 4, Time). Since the Normal‐eyed group spent the shortest WF‐Time, we found that eye‐regressed groups spent a longer in WF‐Time (mean ± SD: Eyeless = 69.92 ± 21.76, Micro‐eyed = 72.49 ± 14.58, Normal‐eyed = 54.42 ± 24.39%, Kruskal–Wallis test: H_2_ = 27.55, *p* < 0.001; Table A5). The average results of WF‐Distance in the eyeless group were the longest, the shortest in Normal‐eyed group and the Micro‐eyed group was in between (mean ± SD: Eyeless 190.18 ± 128.74, Micro‐eyed 156.20 ± 98.79, Normal‐eyed 134.35 ± 105.14 SL, Kruskal–Wallis test: H_2_ = 9.21, *p* < 0.05; Figure 3). Regarding WF‐Frequency, the Eyeless group wall followed most frequently, and the Normal‐eyed group followed the least, with the Micro‐eyed group in between (mean ± SD: Eyeless = 24 ± 16, Micro‐eyed = 22 ± 13, Normal‐eyed = 18 ± 13 times, Kruskal–Wallis test: H_2_ = 8.62, *p* < 0.01). We also found that eyeless species showed the highest WF‐Speed, while the Normal‐eyed group swam at the lowest speed (mean ± SD: Eyeless = 0.52 ± 0.20, Micro‐eyed = 0.45 ± 0.21, Normal‐eyed = 0.36 ± 0.20 SL/s, Kruskal–Wallis test: H_2_ = 22.77, *p* < .001). Since the Eyeless species showed the highest speed, the longest distance and the fastest speed in WF behaviour, they were considered as having the highest intensity of wall‐following. Hence, our results show that WF behaviour in *Sinocyclocheilus* is enhanced with eye degeneration, with the highest enhancement in the Eyeless forms.

### 3.3 Phylogenetic context of wall‐following

The maximum credibility tree of *Sinocyclocheilus* shows four major clades (A–D), as previously reported by Zhao and Zhang (2009) and Mao et al. (2021). The results suggest that wall‐following behaviour is dominant in Clade B, where stygomorphism is greatest (the longest WF‐Distance: *S. microphthalmus* = 222.23 ± 108.86 SL, the longest WF‐Time: *S. tianeensis* 88.41 ± 8.54%, the highest WF‐Speed *S. microphthalmus* 0.67 ± 0.23 SL/s; showed in blue colour, Figure 4). We found that Clade B (mean ± SD = 172.37 ± 113.67 SL) showed significantly higher distance than Clade D (mean ± SD = 128.08 ± 119.75 SL; Dunnett's T3 post‐test: *Z* = 3.50, *p* = .05; Table A6). Clade B also showed the fasted WF‐Speed, the longest WF‐Time and the most WF‐Frequency compared with other clades (Table A6).

However, from Clade C to D, with a predominance of normal sighted species, wall‐following behaviours decreased (the shortest WF‐Distance: *S. longibarbatus* 99.53 ± 74.35 SL, the shortest WF‐Time: *S. purpureus* 44.71 ± 20.52%, the lowest WF‐Speed: *S. longibarbatus* 0.26 ± 0.10 SL/s; Figure 4). But, we found one species to be an outlier to this pattern – *S. macrophthalmus* (Normal‐eyed, stygobitic, Clade C) has a great WF‐Distance as long as the Eyeless cavefishes.

## 4 DISCUSSION

*Sinocyclocheilus* diversification began with the advent of the polar ice caps and the rain‐shadow effect of the Himalayas, as a result of which the Guangxi, Guizhou and Yunnan regions became drier in the late Miocene (Mao et al., 2021). This provided time for diversification across this vast karstic landscape where species acquired, to different degrees and depending on the habitat, many stygomorphic traits. Our study contributes to our understanding of this unfolding of events by revealing wall‐following behaviour as a key evolutionary adaptation within this genus. This behaviour, also appearing in unrelated lineages such as *A. mexicanus* cavefish, paves the way for a deeper exploration of the forces driving this convergent evolution of a stygomorphic behaviour. Therefore, it is crucial to consider the variability within and among clades and the influence of ecological and evolutionary factors on wall‐following behaviour.

Our analysis showed that wall‐following intensity was the highest in Eyeless species and the lowest in Normal‐eyed species. We mainly evaluated three aspects of wall‐following intensity in the *Sinocyclocheilus* genus: time, speed and distance being the most important, as distance is a product of speed and time. However, we also considered speed and time independently, for they can explain more subtle aspects of behaviour (Hoke et al., 2012). We noted the enhancement in wall‐following ability going from normal‐eyed to eyeless species. Eyeless species exhibited the most enhanced wall‐following behaviour (highest speed, distance and a longer time in WF behaviour); however, it is not consistently supported across all measured variables (such as WF‐Time and WF‐Max Speed), even though our analyses (Table A5) confirmed a statistically nonsignificant gradient from Eyeless to Normal‐eyed species for these variables. In our analysis, we retained extreme results, not excluding outliers, as these data points could reveal interesting insights in future in‐depth analyses involving a greater sampling of taxa. Some variations we observed in the data might be attributed to individual differences among species. For instance, the Micro‐eyed species (*S. microphthalmus*) exhibited a greater variability comparable to some Eyeless species, denoting the complexity and diversity within the studied group. Moreover, the term “enhance” does not necessarily imply a linear relationship between the degree of eye development and the magnitude of wall‐following behaviour. It reflects, instead, a behavioural divergence potentially influenced by various factors including sensory capabilities, ecological pressures and phylogenetic history. As such, our data does not strongly support the proposition that Micro‐eyed species represent an “in‐between” state in terms of wall‐following behaviour variables and requires further scrutiny.

The experiments were conducted under near‐dark conditions, as observing and recording cavefish behaviour proved challenging in zero‐lux conditions (due to limitations of the fish‐tracking software). Additionally, while caring for the fish, it was noted that in absolute darkness, the Eyed cavefish ceased movement, becoming immobile; this was the reason for removing these videos following initial trials. Interestingly, most of the cave entrances from which these cavefish were sampled were not in complete darkness, though some species were captured from completely dark environments well within caves (Zhao & Zhang, 2009). Therefore, the light values applied in our experiments corresponded with the natural low‐light conditions observed in the field.

The cave habitats are generally considered to be low in terms of resources and predation pressure (Ajemian et al., 2015; Niemiller & Soares, 2015; Romero et al., 2009). Past studies on cavefish pointed out that swimming behaviour is important in the exploration of habitats in *A. mexicanus* (Teyke, 1985). In addition, wall‐following behaviour is thought to be an extension of swimming for exploration (Sharma et al., 2009). The faster swimming speed of cavefish enables fish to acquire more information via the amplitude of water flow and allows the fish to explore the cave environment continuously (Teyke, 1988). Our findings on wall‐following behaviour is also suggestive of different strategies for resource utilisation and risk avoidance. Continuous swimming behaviour associated with wall‐following, as seen in Eyeless species, may have evolved to optimise resource utilisation in the resource‐poor, predator‐scarce cave environment. Continuous wall‐following has already been shown in *A. mexicanus*, which has been explained in terms of exploratory spatial awareness (Patton et al., 2010; Yoshizawa, 2015). Hence, continuous swimming behaviour associated with wall‐following may have evolved to enable fish to acquire more information via the amplitude of water flow and continuously explore their surroundings to optimise resource utilisation in the near absence of predation. On the contrary, the slower WF‐Speed in Normal‐eyed species might reflect a defensive strategy to reduce the risk of injury. This dual role of wall‐following behaviour in exploration and defence underscores its ecological and evolutionary significance. In other groups, it has been shown that the narrow and fixed wall‐following route could be used as escape routes should threats arise (Ajemian et al., 2015; Ginnaw et al., 2020; Sharma, 2008), while preserving energy to accelerate rapidly when the need arises. In fact, past studies found some other genera of cavefish might need to cope with the risk of predation pressures in cave habitats. For example, cave mollies (*Poecilia mexicana*) were more susceptible to predator attacks within the cave, even in a resource‐rich habitat due to chemoautotrophic primary productivity (Horstkotte et al., 2010; Tobler, 2009; Tobler et al., 2007).

Eyeless species approached stimulation at a narrow angle, while surface‐living species showed a greater approaching distance than cavefish. This could indicate an ability to detect unknown objects earlier, at a longer distance, in Normal‐eyed species, which reflects differences in sensory capabilities and risk assessment strategies among the species. This spatial exploratory behaviour might also be associated with cavefishes' enhanced olfactory and lateral line systems (Chen, Mao, et al., 2022; Fernandes et al., 2018; Kasumyan & Marusov, 2018; Lloyd et al., 2018). Past studies show that in *Sinocyclocheilus* genus, eyed species have more neuromasts compared to eyeless species, which has been interpreted as to the importance of non‐visual sensory expansion to survive in darkness (Chen, Mao, et al., 2022). The lateral line system has evolved to enhance the sensitivity to water flow and enhance sensory‐dependent behaviours to find food more efficiently in *A. mexicanus* cavefish (Espinasa et al., 2023; Lunsford et al., 2022). However, the differences between the visual and non‐visual ability of non‐visual organs still need to be further explored.

Studies on wall‐following behaviour in other lineages highlight different functional explanations, such as risk avoidance in the threespine sticklebacks (Ginnaw et al., 2020) while in Somalian cavefish *Phreatichthys andruzzii*, as an exploratory strategy (Sovrano et al., 2018). Once a behaviour undergoes adaptation to cave environments, it is more likely to persist under relaxed selection pressures, potentially explaining the observed variations in functional explanations across different lineages (Hoke et al., 2012).

Our results also support the enhancement of wall‐following behaviour in stygomorphic species, particularly those in Clade B, even though the patterns are not uniformly clear across all variables or clades. The intricate interplay of genetics, developmental plasticity, ecology and environment possibly shapes the evolution of behavioural traits in these species. The lack of strong phylogenetic correlation in the tested eye‐morphs and behavioural traits in our study may be due to inadequate taxon sampling and the inclusion of species from only one of the clades that contain Eyeless species (Clade B). Due to rarity and sampling‐related problems, we could not analyse wall‐following behaviour of stygomorphic species from Clade D, such as *S. anophthalmus*. However, species within Clade B, namely *S. tianlinensis* and *S. tianeensis*, share a common ancestor; this shared ancestry is suggestive of the evolution of intense wall‐following behaviour in eyeless species due to phylogenetic inertia. Moreover, the evolutionary convergence in the intensity of wall‐following behaviour in unrelated lineages, as observed in our study, supports the idea that this behaviour has evolved in response to similar selective pressures. The prevalence of wall‐following to various degrees across the phylogeny suggests that the trait is ancient.

Due to the rarity of *Sinocyclocheilus* fish and their inaccessibility in deep caves and caverns, it took us 2 years to gather 13 species and a limited number of individuals for the behavioural assay (three individuals from each species). We also had to develop techniques to keep them alive. Given these circumstances, our study included a limited representation of Eyeless and Micro‐eyed *Sinocyclocheilus* species from specific clades. We plan to address this in future studies by expanding our research to encompass a wider range of data from different clades and conducting a deeper analysis.

Finally, our study provides a complex, multi‐faceted picture of wall‐following behaviour in *Sinocyclocheilus* species and its relationship with eye morphology and phylogenetic clades. Low intraspecific variability of wall‐following suggested that this behaviour was fixed for this genus. Some comparable results from other cavefish lineage, such as in *A. mexicanus* cavefishes, show similar wall‐following swimming patterns (Patton et al., 2010). Evolutionary convergence of wall‐following supports the idea that this behaviour has evolved in response to similar selective pressures in evolutionarily unrelated lineages. The prevalence of wall‐following, to various degrees across the phylogeny suggests that the trait is ancient and shared in *Sinocyclocheilus* cavefishes. The insights gained from such research can shed light on the broader patterns and processes of evolution in cave‐dwelling organisms. Future research should continue to explore these relationships, taking into account the inherent variability and complexity of behavioural traits in these fascinating taxon.

## 5 CONCLUSION

Our diversification‐scale behavioural assays show that *Sinocyclocheilus* have wall‐following behaviours associated with cave‐dwelling propensity. The Eyeless species showed the highest intensity of wall‐following behaviour and Normal‐eyed showed the least intensity, with Micro‐eyed forms in between. Our study confirmed that wall‐following is correlated with multiple factors, especially with wall‐following frequency, time and eye‐morphs. Though the determination of the exact function of wall‐following needs further experimentation, we suggest that wall‐following facilitates protection and foraging behaviour in Eyeless forms, and for defence in eyed species. We found that wall‐following is enhanced in Clade B and C (regressed‐eyed species) but reduced in Clade A and D (Normal‐eyed species). However, our results do not show the phylogenetic correlation of wall‐following behaviour, possibly due to inadequate taxon sampling. The convergence of wall‐following with *A. mexicanus* cavefish suggests that this behaviour is an adaptation in response to selective regimes of subterranean environments. Our work will also form the foundation for further cave‐related behavioural work on this emerging multi‐species evolutionary model system.

## AUTHOR CONTRIBUTIONS

**Bing Chen:** Conceptualization (lead); data curation (lead); formal analysis (lead); investigation (lead); methodology (lead); resources (equal); validation (equal); writing – original draft (lead); writing – review and editing (lead). **Wen‐Zhang Dai:** Formal analysis (equal); investigation (equal); methodology (equal); validation (equal); writing – review and editing (equal). **Xiang‐Lin Li:** Data curation (equal); formal analysis (equal); investigation (equal); validation (equal); writing – review and editing (equal). **Ting‐Ru Mao:** Formal analysis (equal); investigation (equal); validation (equal); visualization (equal); writing – review and editing (equal). **Ye‐Wei Liu:** Data curation (equal); investigation (equal); validation (equal); writing – review and editing (equal). **Marcio R. Pie:** Formal analysis (equal); investigation (equal); validation (equal); writing – review and editing (equal). **Jian Yang:** Conceptualization (equal); investigation (equal); supervision (equal); writing – review and editing (equal). **Madhava Meegaskumbura:** Conceptualization (equal); investigation (equal); project administration (lead); resources (lead); supervision (lead); writing – original draft (equal); writing – review and editing (equal).

## FUNDING INFORMATION

This work was supported by the (1) Startup funding for MM through Guangxi University. (2) National Natural Science Foundation of China (#32260333) to MM. (3) National Natural Science Foundation of China (#31860600) to JY for fieldwork. (4) Innovation Project of Guangxi Graduate Education (#YCBZ2021008) to TRM and CB for research work. These funding bodies played no role in the design of the study and collection, analysis and interpretation of data or in the writing of the manuscript.

## CONFLICT OF INTEREST STATEMENT

The authors declare that the research was conducted in the absence of any commercial or financial relationships that could be construed as a potential conflict of interest.

## Supporting information


Video S1.

Video S2.

Video S3.

## Data Availability

The raw data for this article are available at https://datadryad.org/stash/share/vC7HN5DLLLRdWWy9TKQSN95PtjQ‐qXLuSshG23Jqjfc.

## Appendix

**TABLE A1:** *Sinocyclocheilus* species, location, eye morphs, habitat and body shapes of the 13 *Sinocyclocheilus* species tested.

Species	E‐longitude	N‐latitude	Clade	Eye‐morphs	Habitat	Body Shape	Cyt b	ND4
*S. tianlinensis*	106.2900	24.6265	B	Eyeless	Stygobitic	Compressiform	MT373102	MW548431
*S. tianeensis*	107.2036	24.8662	B	Eyeless	Stygobitic	Compressiform	AY854717	AY854774
*S. mashanensis*	108.4447	23.7378	B	Micro‐eyed	Stygophilic	Compressiform	MT373107	MW548430
*S. microphthalmus*	107.0075	24.3102	B	Micro‐eyed	Stygophilic	Compressiform	AY854687	AY854744
*S. bicornutus*	105.1216	25.3141	B	Micro‐eyed	Stygophilic	Compressiform	AY854730	AY854787
*S. multipunctatus*	106.5420	26.4645	C	Micro‐eyed	Stygophilic	Compressiform	AY854712	AY854769
*S. guilinensis*	110.2829	24.9919	A	Normal‐eyed	Stygobitic	Compressiform	MT373104	MW548427
*S. zhenfengensis*	105.6384	25.4773	B	Normal‐eyed	Surface	Compressiform	MK610342	MK610347
*S. macrophthalmus*	107.9398	24.3142	C	Normal‐eyed	Stygophilic	Compressiform	AY854735	AY854792
*S. longibarbatus*	108.0716	25.2921	C	Normal‐eyed	Stygophilic	Fusiform	AY854714	AY854771
*S. oxycephalus*	103.3123	24.7696	D	Normal‐eyed	Stygobitic	Fusiform	AY854685	AY854742
*S. purpureus*	103.6236	23.7720	D	Normal‐eyed	Surface	Compressiform	EU366194	EU366177
*S. maitianheensis*	103.2316	25.0453	D	Normal‐eyed	Surface	Compressiform	AY854710	AY854767
*Cyprinus carpio**	–	–	–	–	–	–	MN544290.1	MN544290.1
*Puntius ticto**	–	–	–	–	–	–	NC_008658	NC_008658
*Labeo batesii**	–	–	–	–	–	–	AB238967	AB238967
*Gymnocypris przewalskii**	–	–	–	–	–	–	AB239595	AB239595
*Gymnocypris eckloni**	–	–	–	–	–	–	AY463522	AY463522

*Note*: GenBank accession numbers of the two mtDNA fragments (ND4 and Cytb) of 13 *Sinocyclocheilus* species and the outgroup. Species name with * were used as the outgroup.

**TABLE A2:** The definition and measurements of all variables in this study.

Variables	Definition	Unit
WF‐Distance	Distance fish swam in wall‐following range/standard length	SL
WF‐Time	Time that fish spend for wall‐following/whole testing time	%
WF‐Resting Time	Time of swimming speeds <0.2 (cm/s)/whole testing time	%
WF‐Speed	Tested by EthoVision XT as average speed	SL/s
WF‐Max Speed	The maximum speed fish reached in one assay	SL/s
WF‐Frequency	The times fish swimming into wall‐following range	–
S‐Time	Time fish swimming in stimulation range	%
S‐Speed	Average speed in stimulation range	SL/s
S‐Max Speed	The max speed in stimulation range	SL/s
S‐Frequency	The times fish swam into stimulation range	–
SE‐diameter	Diameter of eyeball/diameter of eye orbit	–
Standard Length	The length from the tip of mouth to the end of caudal fin	cm
Angle3	Angle of approaching to stimulation, the third time	degree
Angle2	Angle of approaching to stimulation, the second time	degree
Angle1	Angle of approaching to stimulation, the first time	degree
Distance3	Distance of the third approach	cm
Distance2	Distance of the second approach	cm
Distance1	Distance of the first approach	cm

**TABLE A3:** Phylogenetic signal estimated by maximum likelihood with lambda forced to be 0.

Trait	$\hat{\lambda }$	Lik	Lik (*λ* = 0)	*p*‐Value
WF‐Distance	7.25581e‐05	−73.219	−0.00046	1
WF‐Speed	7.25581e‐05	5.744	−0.00040	1
SE‐diameter	7.25581e‐05	−25.687	−0.00042	1
WF‐Frequency	7.25581e‐05	−50.763	−0.00016	1
WF‐Time	7.25581e‐05	−57.643	−0.00049	1
WF‐Max Speed	7.25581e‐05	−23.828	−0.00046	1

**TABLE A4:** The results of approaching angle and distance among the three eye‐morphs.

	Mean ± SD	Median	*N*	Statistical scores	*p*‐Value
Approaching angle					
Stimulation	Wilcoxon rank sum test, *W* = 12,794				.76
3 min‐VB	47.88 ± 23.47	49.83	150		
5 min‐Novel	49.05 ± 25.11	48.14	174		
Eye‐morphs	Kruskal–Wallis rank sum test, H_2_ = 6.43				.04
Eyeless	44.13 ± 21.85	41.97	78	E‐M: *Z* = −0.55	1
Micro‐eyed	46.28 ± 25.92	45.05	90	M‐N: *Z* = −1.78	.23
Normal‐eyed	51.99 ± 24.21	54.77	156	E‐N: *Z* = −2.31	.06
Approaching distance					
Stimulation	Wilcoxon rank sum test, *W* = 13,218				.84
3 min‐VB	1.22 ± 1.15	0.96	150		
5 min‐Novel	1.13 ± 0.99	1.03	174		
Eyemorphs	Kruskal–Wallis rank sum test, H_2_ = 21.72				1.92e‐05
Eyeless	0.72 ± 0.91	0.42	78	E‐M: *Z* = −3.49	1.43e‐03
Micro‐eyed	1.26 ± 1.07	1.12	90	M‐N: *Z* = −0.70	1
Normal‐eyed	1.35 ± 1.08	1.27	156	E‐N: *Z* = −4.5	1.53e‐05

*Note*: Each *p*‐value adjustment method: Bonferroni.

Abbreviations: E, eyeless; M, micro‐eyed; N, normal‐eyed.

**TABLE A5:** The comparison between eye morphs or body shape or 3 different stimuli conditions about every parameter.

	Mean ± SD	Median	*N*	Statistical scores	*p*‐Value
WF‐Distance					
10 min‐No stimulation ANOVA, *F*_2_ = 6.60					.002
*Eye‐morphs*				Tukey's multiple comparisons post‐test	
Eyeless	343.12 ± 78.77	348.4	16	E‐M: diff = −93.51	.03
Micro‐eyed	249.61 ± 99.86	240.33	18	M‐N: diff = −12.58	1
Normal‐eyed	237.03 ± 107.97	219.25	41	E‐N: diff = −106.10	.002
5 min‐Novel stimulation Kruskal–Wallis rank sum test, H_2_ = 16.26					.0003
Eyeless	150.22 ± 84.22	117.13	15	E‐M: *Z* = 1.19	.71
Micro‐eyed	123.39 ± 67.37	102.2	17	M‐N: *Z* = 2.48	.04
Normal‐eyed	78.43 ± 46.5	78.86	40	E‐N: *Z* = 3.76	.001
3 min‐Vibration Attraction Behaviour Kruskal–Wallis rank sum test, H_2_ = 3.19					.20
Eyeless	87.54 ± 30	93.69	18	E‐M: *Z* = −0.59	1
Micro‐eyed	97.04 ± 43.02	93.34	19	M‐N: *Z* = 1.73	.25
Normal‐eyed	77.97 ± 39.12	71.06	35	E‐N: *Z* = 1.02	.92
Body shape Wilcoxon rank sum test, *W* = 2885					.88
Compressiform	152.45 ± 111.91	116	189		
Fusiform	150.85 ± 107.99	119.1	30		
WF‐Speed					
Eye morphs Kruskal–Wallis rank sum test, H_2_ = 22.77					1.14e‐05
Eyeless	0.52 ± 0.2	0.52	49	E‐M: *Z* = 1.70	.27
Micro‐eyed	0.45 ± 0.21	0.4	54	M‐N: *Z* = 2.69	.02
Normal‐eyed	0.36 ± 0.2	0.33	116	E‐N: *Z* = 4.57	1.43e‐05
Stimulation Kruskal–Wallis rank sum test, H_2_ = 17.05					.0001
3 min‐VA	0.47 ± 0.21	0.44	72	3 VA‐5 Novel: *Z* = −3.82	.0003
5 min‐Novel	0.35 ± 0.22	0.33	72	5 Novel‐10 No: *Z* = 3.27	.003
10 min‐No	0.44 ± 0.18	0.44	75	3 VA‐10 No: *Z* = −0.59	1
WF‐Max Speed					
Eye morphs Kruskal–Wallis rank sum test, H_2_ = 21.37					2.29e‐05
Eyeless	3.5 ± 2.54	2.44	49	E‐M: *Z* = −1.15	.74
Micro‐eyed	4.09 ± 2.88	2.66	54	M‐N: *Z* = −3.02	.01
Normal‐eyed	5.1 ± 2.44	4.98	116	E‐N: *Z* = −4.26	6.19e‐05
Stimulation Kruskal–Wallis rank sum test, H_2_ = 4.45					.11
3 min‐VA	11.41 ± 20.1	1.41	72	3 VA‐5 Novel: *Z* = −2.60	.24
5 min‐Novel	20.35 ± 26.35	7.98	72	5 Novel‐10 No: *Z* = −4.38	3.62e‐05
10 min‐No	12.4 ± 20.05	1.07	75	3 VA‐10 No: *Z* = −1.75	.03
WF‐Time					
Eye morphs Kruskal–Wallis rank sum test, H_2_ = 27.55					1.04e‐06
Eyeless	69.92 ± 21.76	73.82	49	E‐M: *Z* = −0.37	1
Micro‐eyed	72.49 ± 14.58	74.76	54	M‐N: *Z* = 4.51	1.92e‐05
Normal‐eyed	54.42 ± 24.39	58.19	116	E‐N: *Z* = 3.94	2.48e‐04
Stimulation Kruskal–Wallis rank sum test, H_2_ = 21.38					2.27e‐05
3 min‐VA	63.45 ± 22.57	69.12	72	3 VA‐5 Novel: *Z* = −2.70	.02
5 min‐Novel	52.24 ± 24.83	54.78	72	5 Novel‐10 No: *Z* = 4.60	1.25e‐05
10 min‐No	70.99 ± 18.41	73.82	75	3 VA‐10 No: *Z* = 1.87	.18
Standard Length					
Eye‐morphs one‐way analysis of means (Welch's ANOVA), *F*_2_ = 1.26					.29
Eyeless	7.77 ± 0.71	7.72	49	E‐M: *Z* = −3.57	1.05e‐03
Micro‐eyed	8.54 ± 1.67	8.7	54	M‐N: *Z* = −0.14	1
Normal‐eyed	8.61 ± 1.2	8.57	116	E‐N: *Z* = −4.28	5.72e‐05
Pectoral fin length					
Eye‐‐morphs Kruskal–Wallis rank sum test, H_2_ = 3.60					.17
Eyeless	1.53 ± 0.2	1.51	49	E‐M: *Z* = −0.35	1
Micro‐eyed	1.59 ± 0.41	1.56	54	M‐N: *Z* = 1.73	.25
Normal‐eyed	1.47 ± 0.31	1.46	116	E‐N: *Z* = 1.27	.62
Body shape Wilcoxon rank sum test with continuity correction, *W* = 5158.5					4.06e‐05
Compressiform	1.55 ± 0.31	1.51	189		
Fusiform	1.28 ± 0.28	1.31	30		
WF‐Frequency					
Eyemorphs Kruskal–Wallis rank sum test, H_2_ = 8.62					.01
Eyeless	24 ± 16	19	49	E‐M: *Z* = 0.27	1
Micro‐eyed	22 ± 13	19	54	M‐N: *Z* = 2.25	.07
Normal‐eyed	18 ± 13	14	116	E‐N: *Z* = 2.48	.04
WF‐Resting Distance					
Eye morphs Kruskal–Wallis rank sum test, H_2_ = 47.07					4.37e‐11
Eyeless	0.05 ± 0.05	0.04	49	E‐M: *Z* = −1.08	.83
Micro‐eyed	0.18 ± 0.29	0.05	54	M‐N: *Z* = −4.99	1.85e‐06
Normal‐eyed	0.89 ± 0.97	0.6	116	E‐N: *Z* = −6.08	3.61e‐09
WF‐Resting Time					
Eyemorphs Kruskal–Wallis rank sum test, H_2_ = 52.724					3.56e‐12
Eyeless	1.44 ± 4.14	0.65	49	E‐M: *Z* = −1.01	.93
Micro‐eyed	3.71 ± 5.38	1.13	54	M‐N: *Z* = −5.33	2.93e‐07
Normal‐eyed	25.39 ± 26.45	16	116	E‐N: *Z* = −6.32	7.40e‐10
Stimulation Kruskal–Wallis rank sum test, H_2_ = 6.60					.04
3 min‐VA	3.44 ± 2.3	3.44	72	3 VA‐5 Novel: *Z* = 2.10	.06
5 min‐Novel	2.98 ± 3.5	1.49	72	5 Novel‐10 No: *Z* = −2.34	1
10 min‐No	5.14 ± 4.1	4.06	75	3 VA‐10 No: *Z* = −0.21	1
S‐Frequency					
Eye‐morphs Kruskal–Wallis rank sum test, H_2_ = 1.41					.50
Eyeless	10 ± 8	8	49	E‐M: *Z* = 0.68	1
Micro‐eyed	7 ± 5	7	54	M‐N: *Z* = 0.41	1
Normal‐eyed	8 ± 8	6	116	E‐N: *Z* = 1.19	.71
S‐Distance					
Eye‐morphs Kruskal–Wallis rank sum test, H_2_ = 1.65					.44
Eyeless	12.07 ± 10.74	8.43	49	E‐M: *Z* = 1.28	.60
Micro‐eyed	8.07 ± 6.91	7.79	54	M‐N: *Z* = −0.75	1
Normal‐eyed	11.28 ± 11.79	6.75	116	E‐N: *Z* = 0.76	1
S‐Time					
Stimulation Kruskal–Wallis rank sum test, H_2_ = 0.55					.76
Eyeless	6.12 ± 7.38	4.63	49	E‐M: *Z* = 0.41	1
Micro‐eyed	4.6 ± 3.43	4.23	54	M‐N: *Z* = 0.27	1
Normal‐eyed	7.06 ± 11.52	3.56	116	E‐N: *Z* = 0.74	1
Stimulation Kruskal–Wallis rank sum test, H_2_ = 3.33					.19
3 min‐VA	6.12 ± 9.05	3.17	72	3 VA‐5 Novel: *Z* = 0.61	1
5 min‐Novel	6.77 ± 12.19	3.83	72	5 Novel‐10 No: *Z* = 1.17	.72
10 min‐No	5.85 ± 5.61	4.7	75	3 VA‐10 No: *Z* = 1.79	.22
S‐Speed					
Eye‐morphs Kruskal–Wallis rank sum test, H_2_ = 1.60					.45
Eyeless	0.68 ± 0.46	0.57	49	E‐M: *Z* = 0.64	1
Micro‐eyed	0.65 ± 0.5	0.53	54	M‐N: *Z* = −1.26	.62
Normal‐eyed	0.89 ± 0.84	0.64	116	E‐N: *Z* = −0.48	1
Stimulation Kruskal–Wallis rank sum test, H_2_ = 18.42					.0001
3 min‐VA	0.95 ± 0.8	0.72	72	3 VA‐5 Novel: *Z* = −4.10	.0001
5 min‐Novel	0.6 ± 0.66	0.36	72	5 Novel‐10 No: *Z* = 3.16	.01
10 min‐No	0.79 ± 0.61	0.63	75	3 VA‐10 No: *Z* = −0.99	.97
S‐Max Speed					
Eye‐morphs Kruskal–Wallis rank sum test, H_2_ = 9.87					.01
Eyeless	4.33 ± 4.66	2.72	49	E‐M: *Z* = 1.70	.27
Micro‐eyed	2.94 ± 3.17	1.91	54	M‐N: *Z* = −3.14	.01
Normal‐eyed	4.11 ± 3.01	3.95	116	E‐N: *Z* = −1.07	.85
Stimulation Kruskal–Wallis rank sum test, H_2_ = 19.52					5.78e‐05
3 min‐VA	3.44 ± 2.3	3.44	75	3 VA‐5 Novel: *Z* = −2.27	.07
5 min‐Novel	2.98 ± 3.5	1.49	72	5 Novel‐10 No: *Z* = 4.42	.00002
10 min‐No	5.14 ± 4.1	4.06	72	3 VA‐10 No: *Z* = 2.13	.10

*Note*: Each *p*‐value adjustment method: Bonferroni.

Abbreviations: No, no stimulation; Novel, novel landscape; S, stimulation; VA, vibration attraction behaviour; Wf, wall‐following.

**TABLE A6:** The comparison between four evolution clades stimuli conditions about every parameter.

	Mean ± SD	Median	*N*	Statistical scores	*p*‐Value
WF‐Distance	Kruskal–Wallis rank sum test, H_2_ = 12.39, df = 3				.006
*Clade*				Dunnett's T3 post‐test	
Clade A	152.82 ± 71.59	132.56	13	A–B: *Z* = 0.11 A–C: *Z* = 1.07 A–D: *Z* = 1.92	1.00 1.00 .33
Clade B	172.37 ± 113.67	128.84	97	B–C: *Z* = 1.73	.50
Clade C	141.22 ± 99.41	110.97	51	C–D: *Z* = 1.34	1.00
Clade D	128.08 ± 119.75	93.5	58	**B–D: *Z* = 3.50**	**.05**
WF‐Speed	Kruskal–Wallis rank sum test, H_2_ = 20.68, df = 3				**.0001**
				Dunnett's T3 post‐test	
Clade A	0.43 ± 0.24	0.35	13	A–B: *Z* = −1.22 A–C: *Z* = 0.33 A–D: *Z* = 1.20	1.00 1.00 1.00
Clade B	0.49 ± 0.2	0.46	97	**B–C: *Z* = 2.68**	**4.40e‐02**
Clade C	0.39 ± 0.19	0.34	51	C–D: *Z* = 1.68	1.00
Clade D	0.34 ± 0.21	0.32	58	**B–D: *Z* = 4.39**	**6.85e‐05**
WF‐Frequency	Kruskal–Wallis rank sum test, H_2_ = 13.63, df = 3				**.003**
				Dunnett's T3 post‐test	
Clade A	19 ± 10	19	13	A–B: *Z* = −0.53 A–C: *Z* = 0.05 A–D: *Z* = 1.47	1.00 1.00 .85
Clade B	22 ± 14	19	97	B–C: *Z* = 1.01	1.00
Clade C	22 ± 16	15	51	C–D: *Z* = 2.27	.14
Clade D	15 ± 13	13	58	**B–D: *Z* = 3.67**	**.001**
WF‐Time	Kruskal–Wallis rank sum test, H_2_ = 28.26, df = 3				**3.20e‐06**
				Dunnett's T3 post‐test	
Clade A	63.64 ± 21.66	69.25	13	A–B: *Z* = −0.82 A–C: *Z* = −0.30 **A–D: *Z* = 2.02**	1.00 1.00 **2.58e‐01**
Clade B	69.52 ± 18.77	71.37	97	B–C: *Z* = 0.87	1.00
Clade C	64.43 ± 23.52	73.56	51	**C–D: *Z* = 3.72**	**1.22e‐03**
Clade D	48.23 ± 24.48	46.81	58	**B–D: *Z* = 5.21**	**1.16e‐06**

*Note*: Each *p*‐value adjustment method: Bonferroni. Bold size showed the parameters wih significant differences.
